# Genome-wide identification and functional analysis of mRNA m^6^A writers in soybean under abiotic stress

**DOI:** 10.3389/fpls.2024.1446591

**Published:** 2024-07-11

**Authors:** Peng Liu, Huijie Liu, Jie Zhao, Tengfeng Yang, Sichao Guo, Luo Chang, Tianyun Xiao, Anjie Xu, Xiaoye Liu, Changhua Zhu, Lijun Gan, Mingjia Chen

**Affiliations:** ^1^ College of Life Sciences, Nanjing Agricultural University, Nanjing, China; ^2^ Department of Criminal Science and Technology, Nanjing Police University, Nanjing, China

**Keywords:** m^6^A, soybean, RNA methylation, abiotic stress, MTA, MTB

## Abstract

N^6^-methyladenosine (m^6^A), a well-characterized RNA modification, is involved in regulating multiple biological processes; however, genome-wide identification and functional characterization of the m^6^A modification in legume plants, including soybean (*Glycine max* (L.) Merr.), remains lacking. In this study, we utilized bioinformatics tools to perform comprehensive analyses of molecular writer candidates associated with the RNA m^6^A modification in soybean, characterizing their conserved domains, motifs, gene structures, promoters, and spatial expression patterns. Thirteen m^6^A writer complex genes in soybean were identified, which were assigned to four families: MT-A70, WTAP, VIR, and HAKAI. It also can be identified that multiple cis elements in the promoters of these genes, which were classified into five distinct groups, including elements responsive to light, phytohormone regulation, environmental stress, development, and others, suggesting that these genes may modulate various cellular and physiological processes in plants. Importantly, the enzymatic activities of two identified m^6^A writers, GmMTA1 and GmMTA2, were confirmed *in vitro*. Furthermore, we analyzed the expression patterns of the *GmMTA*s and *GmMTB*s under different abiotic stresses, revealing their potential involvement in stress tolerance, especially in the response to alkalinity or darkness. Overexpressing *GmMTA2* and *GmMTB1* in soybean altered the tolerance of the plants to alkalinity and long-term darkness, further confirming their effect on the stress response. Collectively, our findings identified the RNA m^6^A writer candidates in leguminous plants and highlighted the potential roles of *GmMTA*s and *GmMTB*s in the response to abiotic stress in soybean.

## Introduction

Over 150 distinct chemical modifications of eukaryotic RNA molecules have been identified, including methylation, acetylation, and glycosylation (Delaunay et al., 2023). Many exist in noncoding RNAs, particularly transfer RNAs and ribosomal RNAs. Massage RNA (mRNA) can also carry several base modifications, such as N^6^-methyladenosine (m^6^A), N^1^-methyladenosine, 5-methylcytidine, N^4^-acetylcytidine, N^7^-methylguanosine, and pseudouridine (Frye et al., 2016, 2018). These chemical modifications influence gene expression by regulating the structure, splicing, transport, stability, and translation efficiency of the target transcripts (Roundtree et al., 2017). Of all known modifications, m^6^A is the most abundant in mRNA (Jia et al., 2013). It is installed by a “writer” protein complex and dynamically removed by “eraser” proteins in the nucleus (Shi et al., 2019). Often, “reader” proteins are responsible for decoding the m^6^A signature (Han et al., 2021).

In plants, m^6^A modifications in mRNA are decorated by a methyltransferase complex comprising MRNA ADENOSINE METHYLASE A (MTA; the ortholog of human METHYLTRANSFERASE-LIKE 3 (METTL3), MRNA ADENOSINE METHYLASE B (MTB; the ortholog of human METTL14), FKBP12 INTERACTING PROTEIN 37 kDa (FIP37), VIRILIZER (VIR), and the E3 ubiquitin ligase HAKAI (Zhong et al., 2008; Shen et al., 2016). Mutations of *MTA*, *MTB*, *FIP37*, or *VIR* in Arabidopsis (*Arabidopsis thaliana* (L.) Heynh.) are embryo-lethal, indicating that m^6^A RNA modifications are essential for plant development (Vespa et al., 2004; Zhong et al., 2008; Shen et al., 2016; Růžička et al., 2017). The absence of HAKAI decreases the abundance of m^6^A modifications but does not result in obvious growth defects (Růžička et al., 2017). In cotton (*Gossypium hirsutum* (L.)), m^6^A modifications enhance the stability of *GhMYB44* mRNA, contributing to fiber elongation and secondary cell wall thickening (Xing et al., 2023). m^6^A modifications in plants are also involved in the stress response. In Arabidopsis, transcripts involved in the salt and osmotic stress responses showed an increased abundance of m^6^A modifications when the plants were grown under high salinity (Anderson et al., 2018). In apple (*Malus domestica* (Suckow) Borkh.), MdMTA-mediated m^6^A modifications improved drought tolerance by promoting the mRNA stability and translation efficiency of genes associated with lignin deposition and oxidative stress (Hou et al., 2022). The overexpression of the m^6^A reader *Malus hupehensis* YTH-domain family protein 2 (MhYTP2) can elevate the mRNA stability of its target *M. domestica* allantoinase-like gene (*MdALN*) in apple (Guo et al., 2023). Together, this evidence highlights the significant roles of m^6^A modifications in developmental regulation and stress tolerance in plants.

Soybean (*Glycine max* (L.) Merr.) is an economically important leguminous crop, as it contributes a huge proportion of the total global oilseed and biodiesel and provides vital protein and oil sources for human food and animal fodder (Qi and Lee, 2014; Kim et al., 2017). Soybean cultivation faces challenges from abiotic stresses, such as high or low temperatures and soil salinity or alkalinity. Over the course of its long evolutionary history, soybean has developed intricate strategies to withstand abiotic stresses, which involve modifications across multiple dimensions, including its metabolism, physiology, and transcriptome (Feng et al., 2021; Sheikh et al., 2024). One such abiotic stress, soil salinity, causes ion toxicity and osmotic stress, and thus induces the generation of reactive oxygen species (ROS) (van Zelm et al., 2020). Enzymatic antioxidants, such as superoxide dismutase (SOD), peroxidase (POD), and catalase (CAT), can remove ROS to reduce oxidative stress and protect plants from damage (Wang et al., 2016a; Fu et al., 2017). Additionally, ion channel proteins play a crucial role in the response to salt stress; for instance, Na^+^/H^+^ ANTIPORTER 1 (GmNHX1) sequesters Na^+^ into the vacuole, thereby reducing its level in the cytoplasm (Yang et al., 2017). Similarly, CATION/PROTON EXCHANGER 1 (GmCHX1) facilitates the exclusion of Na^+^ from leaf tissues, mitigating the toxic effects associated with excessive salt accumulation (Qu et al., 2022). Moreover, stress-inducible transcription factors (TFs), including NACs, bZIPs, MYBs, and WRKYs (Katiyar et al., 2012; Ji et al., 2013; Zhu et al., 2014; Phukan et al., 2016; Lim et al., 2022), enhance plant stress tolerance by regulating the expression of target genes during the stress response. Gene expression is also modulated by epigenetic modifications, including m^6^A, which play important roles in plant responses to abiotic stress; however, studies on the involvement of m^6^A in the soybean response to abiotic stress are limited, and the key components involved have yet to be identified.

In this study, we employed genome-wide analyses to identify the m^6^A writer proteins in soybean and analyzed the gene structure and evolutionary aspects of each family member. We particularly focused on elucidating the expression patterns and subcellular localization of the core members of the m^6^A writer complex, the GmMTAs and GmMTBs, which were determined to be involved in abiotic stress responses. Our study provides a foundation for further exploring the function of the m^6^A modification in soybean.

## Materials and methods

### Plant materials and stress treatments

Soybean (*Glycine max*, Williams 82) seeds were sterilized using chlorine and then planted on moist vermiculite. The plants were cultivated in a growth chamber under long-day conditions (16 h light of 100 μmol m^-2^ s^-1^ intensity provided by white LED lamps at 25°C and 8 h dark at 23°C, 70% relative humidity). 15-day-old seedlings were used for the different abiotic stress treatment. For the cold or heat treatments, seedlings were grown at 8°C or 42°C for 24 h, respectively. For the drought stress treatment, soybean seedlings were transferred to Hoagland liquid culture containing 20% polyethylene glycol (PEG) and incubated for 1 day. For the salinity or alkalinity stress treatments, soybean plants were grown in Hoagland liquid culture containing 150 mM NaCl or 100 mM NaHCO_3_, respectively, for 1 day. For the darkness treatment, soybean seedlings were grown in Hoagland liquid culture under the dark condition for 3 days. Following these abiotic stress treatments, the leave and roots samples were collected separately and frozen at -80°C for subsequent experiments.

### Soybean leaf transient transformation

To generate transgenic soybean lines overexpressing *GmMTA2* or *GmMTB1*, a modified soybean transient transformation was utilized based on a previously study (Wang et al., 2023a). In brief, the transformed *Agrobacterium* cells were incubated with infiltration buffer (OD_600_ = 1) containing 10 mM MES (pH 5.6) and 200 μM acetosyringone (Sigma) and infiltrated into the lower epidermis of the leaves from 7-day-old soybean seedlings by employing a vacuum pump until the leaves were thoroughly wetted. Following a recovery period of one day under the continuous darkness, the transformed soybean seedlings were transferred to the normal growth condition for another five days before being subjected to the stress treatment. For alkalinity treatment, the seedlings were grown in Hoagland liquid culture containing 100 mM NaHCO_3_ for 36 hours. Histochemical detection of hydrogen peroxide (H_2_O_2_) and superoxide anion (O^2.-^) and the enzymatic activity measurement of CAT, POD, and SOD from the infiltrated leaves were performed according to previous studies (Cheng et al., 2019; Pi et al., 2023). For darkness treatment, the seedlings were grown under the continuous darkness for 10 days. Chlorophyll content of soybean leaves from each sample was measured according to a previous description (Li et al., 2024).

### RNA extraction, cloning, and quantitative RT-PCR analyses

Total RNA was isolated from 15-day-old soybean leaves and cDNA was prepared according to previous studies (Anderson et al., 2018; Gao et al., 2023; Wang et al., 2023b). The following primers were used for cloning: for *GmMTA1* (Glyma.07G067100), Cp994 and Cp995; for *GmMTA2* (Glyma.16G033100), Cp1293 and Cp1294, for *GmMTB1* (Glyma.10G232300), Cp988 and Cp989; for *GmMTB2* (Glyma.20G161800), Cp1291 and Cp1292 (**Supplementary Table S3**). *GmMTA1*, *GmMTA2*, *GmMTB1*, and *GmMTB2* were cloned into pBA002-flag-HA-StrepII (VN21) and pXCS-YFP (V36) (Chen and Witte, 2020), respectively, for the protein purification and subcellular localization analysis. The VN21 was generated by introducing a 135-bp fragment encoding the flag-HA-StrepII tag using the primer pair Cp795/Cp796 into pBA002 vector (Kost et al., 2008).

To analyze mRNA abundance, reverse transcription quantitative PCR (RT-qPCR) was performed with QuantStudio 1 (Thermo Fisher Scientific) using Hieff qPCR SYBR Green Master Mix (Yeasen Biotechnology) according to the previous description (Wang et al., 2023b). Total RNA from each sample was isolated using the RNA isolater Total RNA Extraction Reagent (Vazyme Biotech Co., Ltd). The quantity and concentration of the resulted RNA were evaluated using a Thermo Scientific NanoDrop™ spectrophotometer. A total of 800 ng of RNA was used to synthesize the first-strand cDNA using an Oligo (dt) primer. Transcript abundance of *GmMTA1*, *GmMTA2*, *GmMTB1*, and *GmMTB2* was analyzed by employing the primer pairs Cp633/Cp634 or Cp631/Cp632, Cp637/Cp638, and Cp635/Cp636, respectively. *GmF-BOX* (Glyma.12G051100) was amplified with the primer pairs Cp363/Cp364 as an internal reference gene in mRNA. The calculation was based on the 2^−ΔΔCT^ method (Livak and Schmittgen, 2001). All primer sequences are detailed in **Supplementary Table S3**.

### Phylogenetic and characteristics analyses of the components m^6^A writer proteins in soybean

The protein sequences of Arabidopsis m^6^A writers were obtained from the TAIR database (https://www.arabidopsis.org) and used as reference sequences. Subsequently, the m^6^A writer protein sequences from *Glycine max*, *Glycine soja, Phaseolus vulgaris, Medicago truncatula*, and *Lotus japonicus* were identified by using the BlastP method from Phytozome database (https://phytozome-next.jgi.doe.gov). Multiple sequence alignment was performed with the ClustalW method (Larkin et al., 2007). To compare evolutionary relationships, the m^6^A writer protein sequences from *A. thaliana*, *G. max*, *G. soja*, *P. vulgaris*, *M. truncatula*, and *L. japonicus* were used to construct a phylogenetic tree using MEGA11 (Tamura et al., 2021) with the Neighbor-Joining (NJ) method and 1000 bootstrap replications. Thereafter, the phylogenetic tree was visualized using ChiPlot (https://www.chiplot.online/) (Xie et al., 2023).

### Analyses of gene structure, conserved motifs, collinearity relationship, and cis-elements analyses of the m^6^A writer genes

Gene structures were visualized using the TBtools software with GFF files provided as input. Conserved motifs within the m^6^A writer protein sequences were analyzed using the MEME online tool (https://meme-suite.org/meme/tools/meme). The parameters were set as following: the site distribution was designated as ‘any number of repetitions’ (anr), the number of motifs was specified as 10, and all other optional parameters were kept at their default settings. Results of the conserved domains were visualized by using TBtools. To investigate the collinearity relationships among m^6^A writer genes in *G. max*, the One Step MCScanX-Super Fast program integrated into TBtools was employed. For cis-elements analysis, 2000-bp region upstream of the start codons of m^6^A writer genes were obtained from the Phytozome database. Subsequently, the promoter sequences were submitted to the PlantCARE database (https://bioinformatics.psb.ugent.be/webtools/plantcare/html/). Prediction from the PlantCARE database were visualized using TBtools (Chen et al., 2020).

### Tissue-specific expression of the m^6^A writer proteins in soybean

To analyze the tissue-specific expression of m^6^A writer proteins, transcriptome sequencing data were obtained from the SoyOmics database (Liu et al., 2023) for various soybean tissues, including cotyledon, stem, leaf bud, leaf, flower, seed, shoot, and root. These data were graphically represented and visualized using the ChiPlot.

### Subcellular localization

GmMTAs-eYFP or GmMTBs-eYFP was transiently co-expressed with the nucleus marker protein RFP-H2B (RFP fused to histone 2B) (Martin et al., 2009) in *Nicotiana benthamiana* leaves for 5 days. The samples were analyzed using a ZEISS LSM 980 with Airyscan2 microscope equipped with an HC PLAPO CS2 40 × 1.0 water immersion objective (ZEISS Microsystems) according to the previous description (Wang et al., 2023b).

### Protein purification and enzymatic activity measurement

Recombinant soybean GmMTA1 and GmMTA2 were affinity purified after transient expression in *N. benthamiana* as described before (Chen et al., 2016; Baccolini and Witte, 2019). Purified protein was quantified by employing the Bradford reagent from Tiangen with bovine serum albumin (BSA) as the standard.

To assess the enzymatic activity, 0.15 nmol of recombinant GmMTA1 or GmMTA2 was incubated with a 50 µl substrate solution, containing 0.8 mM S-adenosylmethionine (SAM; Sigma), 0.15 nmol RNA probe (UACACUCGAUCUGGACUAAAGCUGCUC, synthesized by Genscript), 80 mM KCl, 1.5 mM MgCl_2_, 0.2 U μl^−1^ RNasin, 10 mM dithiothreitol (DTT), 4% glycerol and 15 mM HEPES (pH 7.9), at 28°C for 1 h. Then the reaction was terminated by incubating at 95°C for 15min. The resulting RNA product (800 ng) were fully digested into single nucleosides in a 50μl reaction buffer containing 10 mM Tris–HCl, pH 7.9, 1 mM MgCl_2_, 0.1 mg mL^−1^ BSA, 0.4 units benzonase (Sigma-Aldrich), 0.004 units phosphodiesterase I (Sigma-Aldrich) and 0.04 units shrimp alkaline phosphatase (NEB) according to previous description with minor revision (Chen et al., 2018; Chen and Witte, 2020; Gao et al., 2023; Wang et al., 2023b). After incubation at 37°C for 10 h, enzyme reaction was terminated and the sample was filtered by an ultrafiltration tube (3 kDa cutoff; Pall). 2μl aliquots were analyzed by an Agilent 1290 HPLC system coupled with a Sciex 6500 Qtrap mass spectrometer. The following mass transitions were monitored: m/z 268.1 to 136 (A, adenosine); m/z 282.12 to 150 (m^6^A, N6-methyladenosine). Standard solutions of A: 1, 5, 25 50, 100, 200, 400, 2000 and 10 000 ng/ml; m^6^A: 0.1, 0.5, 2.5, 5, 10, 20, 40, 200, and 1000 ng/ml were used for quantification. The ratio of m^6^A to A were calculated based on the calibrated concentrations.

### Statistics

Statistical analysis was performed by GraphPad Prism 9.5.1 software. Statistical methods and sample sizes are shown in the figure legends. All replicates are biological replicates or experimental replicates.

## Results

### Genome-wide identification and evolutionary analysis of mRNA m^6^A writer genes in legume plants

**Figure 1:**
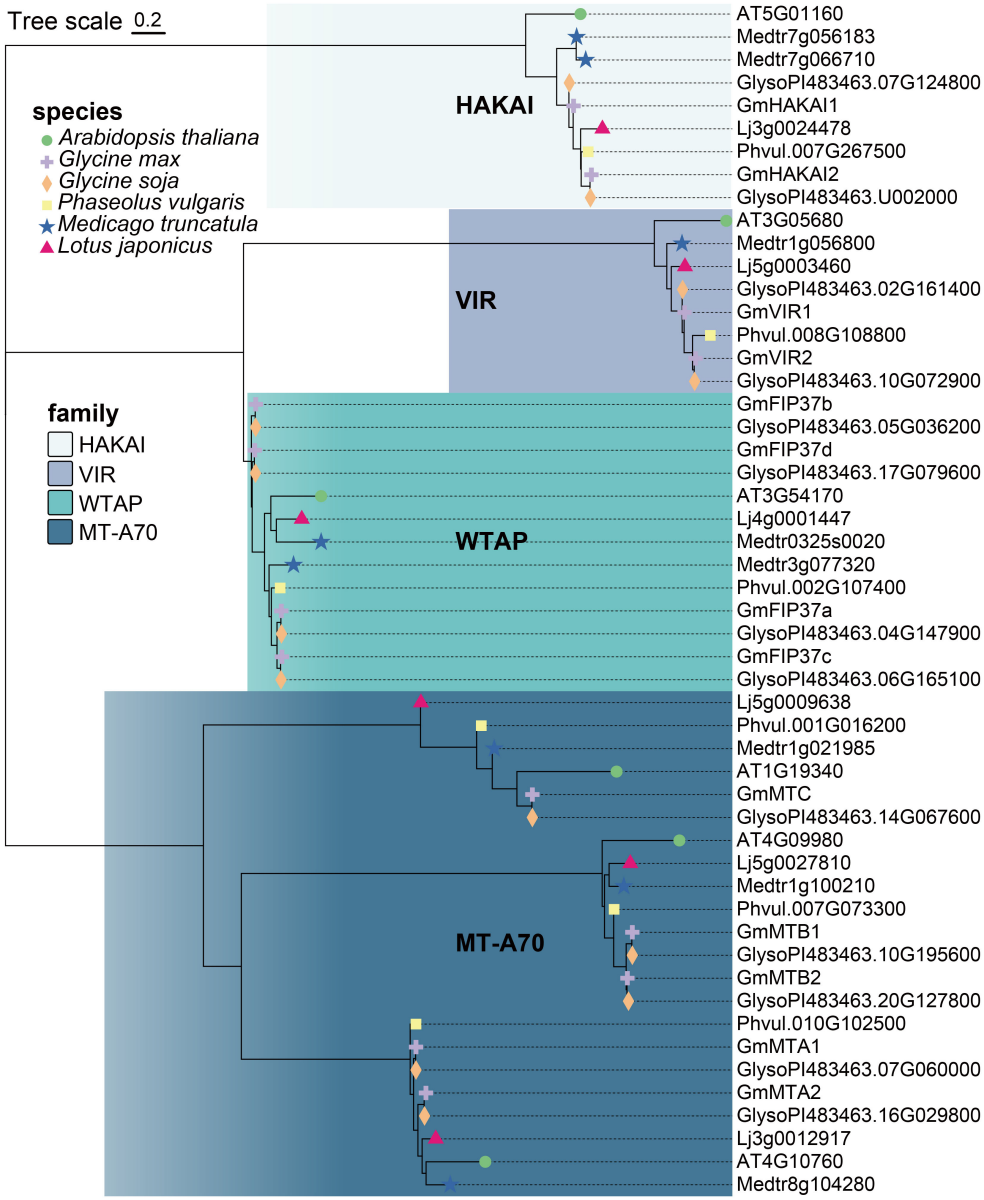
Phylogenetic analysis of m^6^A writer proteins in *Arabidopsis thaliana*, *Glycine max*, *Glycine soja*, *Phaseolus vulgaris*, *Medicago truncatula*, and *Lotus japonicus.* The phylogenetic tree was constructed using MEGA11 software with the Neighbor-Joining algorithm and 1,000 bootstrap replicates.

To identify the m^6^A writer candidate genes in legume plants, we used the sequences of the known writer proteins MTA, MTB, MTC, FIP37, VIR, and HAKAI from the model plant Arabidopsis as queries in BLASTp searches against the genomes of soybean (*G. max*), wild soybean (*G. soja* Siebold & Zucc.), common bean (*Phaseolus vulgaris* L.), *Medicago truncatula* Gaertn., and *Lotus japonicus* (Regel) K. Larsen in the Phytozome V13 database (https://phytozome-next.jgi.doe.gov/blast-search). We identified thirteen candidate m^6^A writer homologs in *G. max*, thirteen in *G. soja*, six in *P. vulgaris*, eight in *M. truncatula*, and six in *L. japonicus* (**Figure 1**; **Supplementary Table S1**). All identified proteins were divided into four families (MT-A70, WTAP, VIR, and HAKAI) according to their topological structure. At least one homolog for each family was identified in each of the plant species, with the MT-A70 family representing the most identified (22 candidates in total) and the least identified for the VIR family (eight in total). Among the five analyzed legumes, the *G. max* and *G. soja* genomes contained the highest number of m^6^A writer candidate genes (**Supplementary Table S1**). In *G. max*, five candidate genes were identified in the MT-A70 family, including *Glyma.14G077000*, *Glyma.10G232300*, *Glyma.20G161800*, *Glyma.07G067100*, and *Glyma.16G033100*. Four candidate genes were identified for the WTAP family, and two each were revealed for the VIR or HAKAI families (**Figure 1**; **Table 1**). The members of each writer protein family were named sequentially according to their order on the chromosomes (**Table 1**).

**Table 1:** Characteristics of predicted m^6^A writer candidate genes in *Glycine max*.

Family	Gene name	Gene ID (Phytozome)	Amino acid length	Isoelectric point	Molecular weight (kDa)	Subcellular localization prediction	Orthologous gene ID in *A. thaliana*
MT-A70	*GmMTA1*	Glyma.07G067100	762	6.22	84.61	nucleus	AT4G10760
	*GmMTA2*	Glyma.16G033100	761	5.95	84.26	nucleus	
	*GmMTB1*	Glyma.10G232300	1102	6.65	121.73	nucleus	AT4G09980
	*GmMTB2*	Glyma.20G161800	1098	6.76	121.47	nucleus	
	*GmMTC*	Glyma.14G077000	428	7.14	48.88	nucleus	AT1G19340
WTAP	*GmFIP37a*	Glyma.04G186400	354	5.75	40.34	nucleus	AT3G54170
	*GmFIP37b*	Glyma.05G040200	339	5.12	38.34	nucleus	
	*GmFIP37c*	Glyma.06G179400	343	5.59	39.17	nucleus	
	*GmFIP37d*	Glyma.17G086600	343	5.04	38.75	nucleus	
VIR	*GmVIR1*	Glyma.02G195600	2230	5.36	246.01	nucleus	AT3G05680
	*GmVIR2*	Glyma.10G082100	2174	5.35	239.33	nucleus	
HAKAI	*GmHAKAI1*	Glyma.07G144300	439	6.2	47.89	nucleus	AT5G01160
	*GmHAKAI2*	Glyma.18G195500	440	6.15	47.94	nucleus	

We identified similar characteristics in the candidate proteins from each family in *G. max.* The lengths of the MT-A70 family candidate proteins ranged from 428 to 1102 amino acids. The predicted molecular weights were 48.88 to 121.73 kDa, with theoretical isoelectric points (pIs) in the range of 5.95 to 7.14. The two VIR family candidates had the longest sequences, which were 2230 and 2174 aa with molecular weights of 246.01 and 239.33 kDa and pIs of 5.36 and 5.35. The HAKAI and WTAP candidates were shorter, ranging from 439 to 440 aa and 343 to 354 aa, respectively. Their molecular weights ranged from 47.89 to 47.94 kDa (HAKAI family) and 38.34 to 40.34 kDa (WTAP family), and their pIs were 6.15 to 6.2 and 5.04 to 5.75, respectively.

### Conserved motifs and gene exon–intron structures of the mRNA m^6^A writer genes

**Figure 2:**
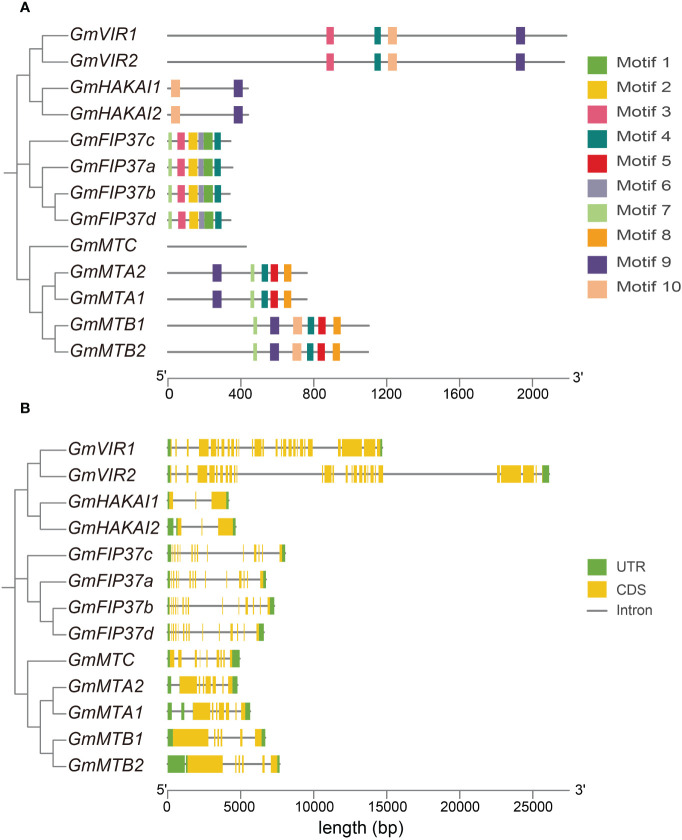
The conserved motifs and gene structure of m^6^A writer genes in *G max*. **(A)** Phylogenetic analysis of m^6^A writer candidates from *G max* and the organization and distribution of the conserved motifs in the m^6^A writer genes. **(B)** Phylogenetic analysis of m^6^A writer candidates from *G max* and the exon–intron structures of the m^6^A writer genes. Untranslated regions (UTRs) are represented by green boxes, coding sequences (CDSs) are represented by yellow boxes, and introns are represented by gray lines.

We constructed a phylogenetic tree using the Neighbor-Joining method to reconstruct the evolutionary relationships among all writer candidates from *G. max* (**Figure 2**). The result was consistent with that of the phylogenetic analysis constructed using the proteins from all five legume plants and Arabidopsis (**Figure 1**). We employed a motif analysis using the MEME program to identify the conserved motifs present within the m^6^A writer candidates in *G. max*. In total, 10 distinct and highly conserved motifs were predicted (**Figure 2A**; **Supplementary Table S2**). Each candidate protein contained 2–6 motifs except GmMTC (**Figure 2A**), hinting that most are likely m^6^A writer proteins. Motif 4, motif 7, and motif 10 were common to many members, suggesting that they may be important for methyltransferase activity. Additionally, motif 1, motif 2, and motif 3 were unique to the WTAP/FIP37 subfamily, while motif 5 and motif 8 exclusively existed in the MT-A70 subfamily. The homologous proteins of each family share identical conserved motifs, suggesting they may be functionally redundant.

We analyzed the exon–intron distribution to investigate the genetic structural diversity. Notably, m^6^A writer candidates from each subfamily had a similar exon–intron pattern, although across all writer candidates the exon number varied substantially, from 3 to 28. The two *GmVIR*s had the most exons (27 and 28 exons), while two *GmHAKAI*s had three exons each. Seven and six exons, respectively, were identified in the core methyltransferase genes, *GmMTA*s and *GmMTB*s. Generally, closely related candidate writers tended to have similar conserved motifs (**Figure 2A**) and exon–intron structure patterns (**Figure 2B**), suggesting their relative conservation during the evolutionary process and thereby substantiating the accuracy of the clustering analysis presented in **Figure 1**.

### Chromosomal distribution of mRNA m^6^A writer genes

**Figure 3:**
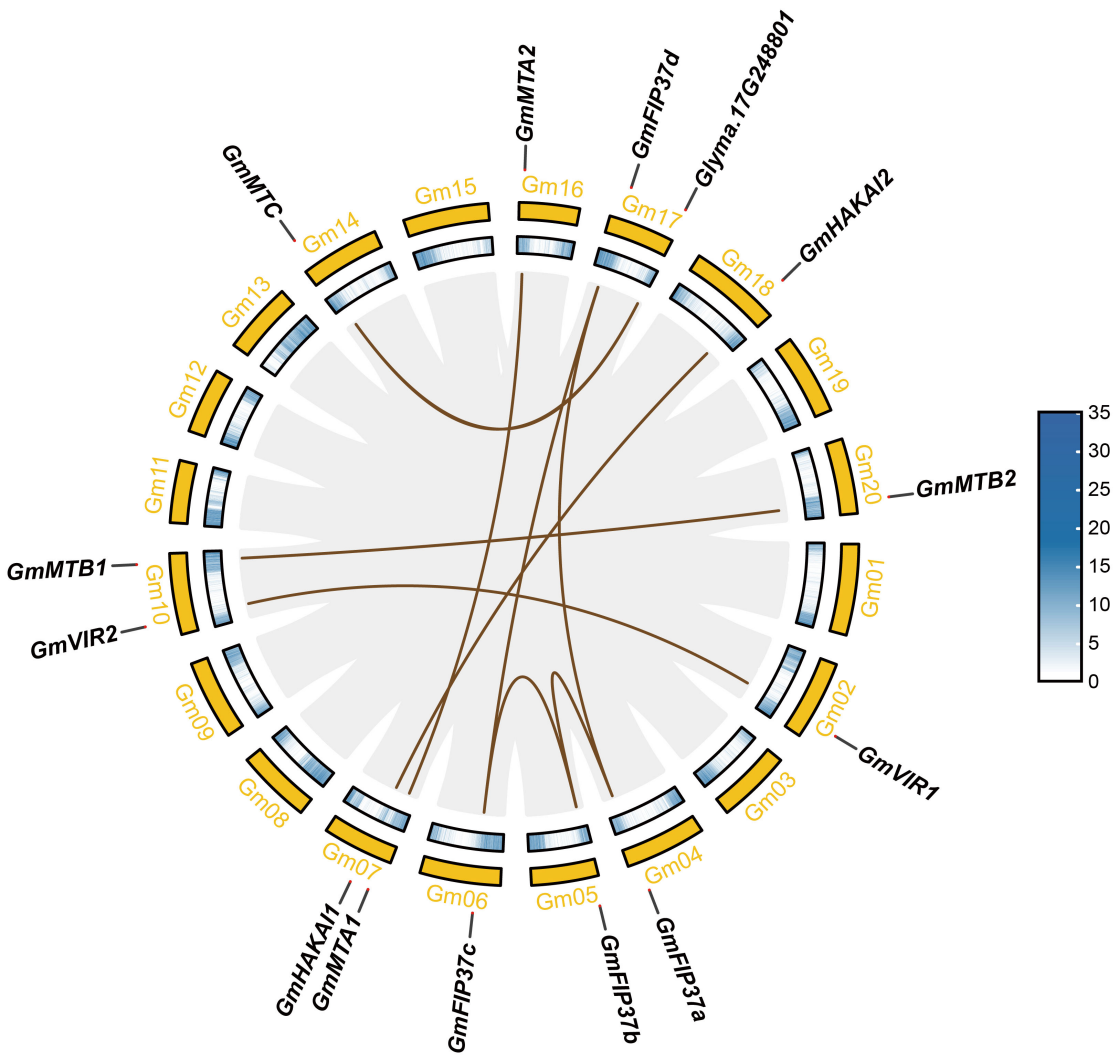
Collinearity analysis of the m^6^A writer genes in soybean. The tan lines represent collinear pairs of m^6^A writer genes, and the gray lines represent the collinearity results of the soybean genome.

We investigated the chromosomal distribution of the m^6^A writer genes in soybean and identified associated gene duplication events. In total, 13 genes were randomly distributed on 11 of the 20 chromosomes of *G. max* (**Figure 3**). Chromosomes 7 and 10 each possess two m^6^A writer genes, whereas chromosomes 2, 4, 5, 6, 14, 16, 17, 18, and 20 each contain one such gene. Different gene replication events, such as tandem duplication and fragment duplication, occur in plant genomes, resulting in the expansion of gene families (Cannon et al., 2004). The collinearity analysis revealed no tandem duplication between the m^6^A writer genes; however, nine pairs arising from fragment duplications (*GmMTA1*/*GmMTA2*, *GmMTB1*/*GmMTB2*, *GmMTC*/*Glyma.17G248801*, *GmVIR1*/*GmVIR2*, *GmHAKAI1*/*GmHAKAI2*, *GmFIP37a*/*GmFIP37b*, *GmFIP37b*/*GmFIP37c*, *GmFIP37c*/*GmFIP37d*, and *GmFIP37a*/*GmFIP37d*) were observed, showing that members from each m^6^A writer family originated from gene duplication in soybean. A duplication partner from one such pair, *Glyma.17G248801*, is an uncharacterized gene without an MT-A70 domain, suggesting that it possesses an unknown function other than m^6^A methyltransferase activity.

### Spatial expression patterns and subcellular localization of the mRNA m^6^A writer genes and proteins

Employing the publicly available data from the SoyOmics website (https://ngdc.cncb.ac.cn/soyomics/index), we examined the expression profiles of all m^6^A writer genes across various tissues from *G. max*, including the cotyledon, stem, leaf bud, leaf, flower, pod_seed (pod plus seed), pod, seed, shoot meristem, and root. Our analyses revealed distinct expression patterns among these genes across different tissue types (**Supplementary Figure S1**). Notably, we observed a pronounced upregulation of the expression of m^6^A writer genes in the leaf bud, flower, and shoot, indicating their potential involvement in orchestrating floral development. By contrast, we were surprised to discover relatively low expression levels of these genes in the leaves.

**Figure 4:**
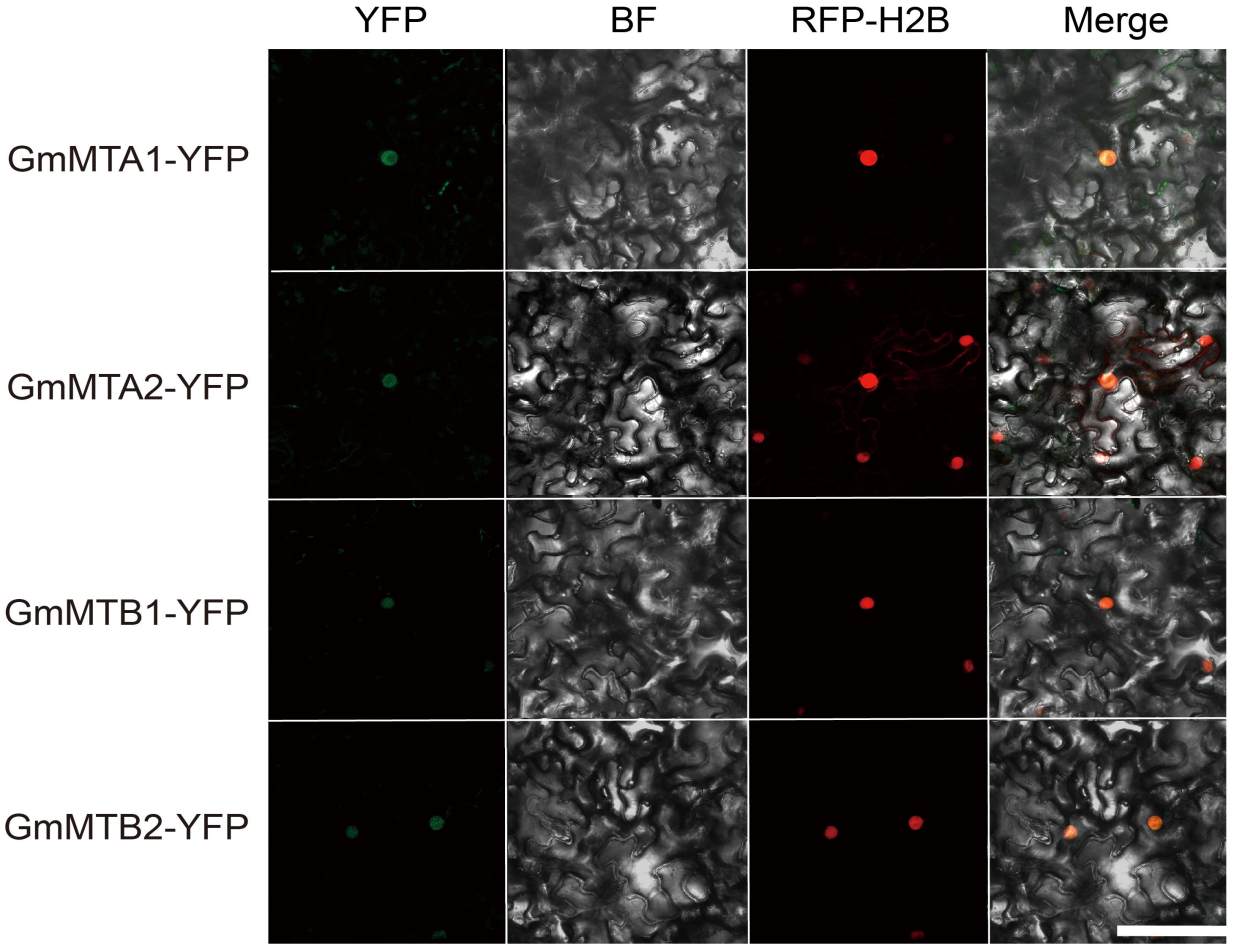
Subcellular localization analysis of GmMTAs and GmMTBs in *N. benthamiana* leaves. RFP-H2B was used as a nuclear marker. Bar, 100 μM. BF, brightfield.

Furthermore, we used WoLF PSORT (https://wolfpsort.hgc.jp/) to predict the subcellular localization of all members, which were all predicted to be localized in the nucleus (**Table 1**). To test this prediction, we fused the full-length amino acid sequences of the four core members, GmMTA1, GmMTA2, GmMTB1, and GmMTB2, with a C-terminal yellow fluorescent protein (YFP) tag each and then independently transiently co-expressed them with the nucleus marker (H2B) fused with mCherry (RFP) in *N. benthamiana* leaves. The results showed that all four proteins were indeed localized to the nucleus (**Figure 4**), which is consistent with the prediction.

### Methyltransferase activity analysis of the GmMTAs

**Figure 5:**
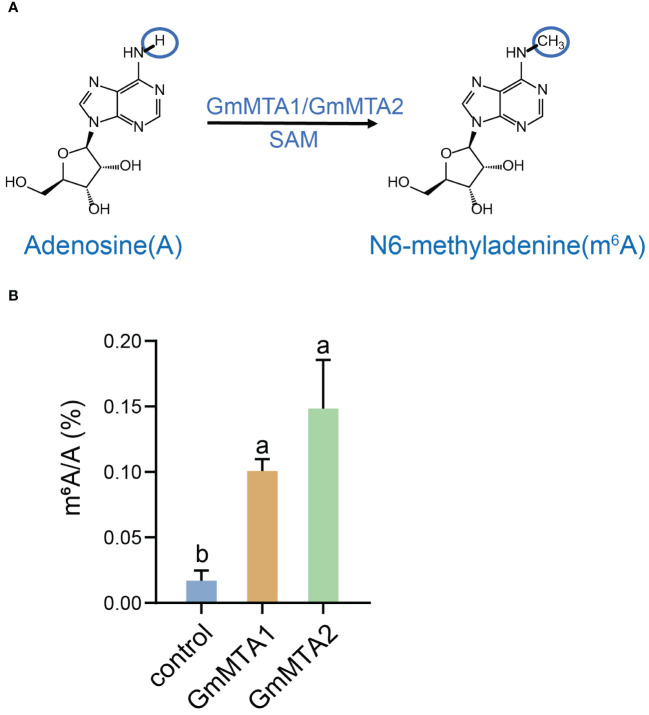
Biochemical analyses of GmMTA1 and GmMTA2. **(A)** A proposed reaction mechanism of adenosine methylation producing the N^6^-methyladenosine (m^6^A) RNA modification performed by GmMTAs in soybean. **(B)** *In vitro* methylation assay of GmMTA1 and GmMTA2. Error bars represent SD (*n* = 3). Different letters indicate significant differences at *P* < 0.05.

In mammals, an MTase complex comprising METTL3 and METTL14 efficiently catalyzes the transfer of a methyl group to m^6^A on RNA. METTL3 primarily functions as the catalytic core, while METTL14 acts as an RNA-binding platform (Wang et al., 2016b). To investigate the enzymatic activities of GmMTAs, which are the homologs of mammalian METTL3 in soybean, we independently expressed *GmMTA1* and *GmMTA2* recombinantly in *N. benthamiana* and performed methylation assays with the purified proteins using an unmodified RNA probe and SAM as the methyl donor (**Figure 5A**). Subsequently, the RNA product was digested into nucleosides and the abundance of m^6^A was analyzed using liquid chromatography–tandem mass spectrometry (LC-MS/MS). Remarkably, our findings revealed a significant increase in m^6^A levels upon adding GmMTA1 or GmMTA2 proteins compared with the control. These experimental results strongly support our hypothesis that GmMTA1 and GmMTA2 possess similar methyltransferase activities, indicating their ability to catalyze mRNA m^6^A modifications (**Figure 5**).

### Cis-element analyses of mRNA m^6^A writer genes

**Figure 6:**
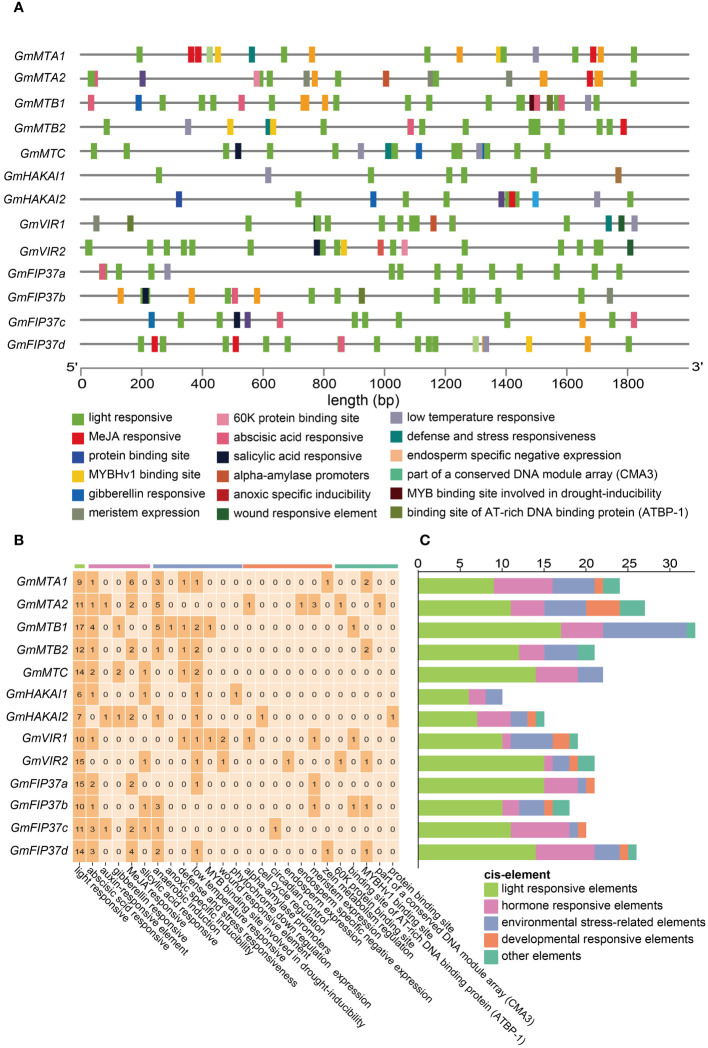
Cis elements in the promoters of m^6^A writer genes. **(A)** The distribution of cis elements in the promoters of the m^6^A writer genes. Different cis elements were depicted in different colored boxes. **(B)** The number of each category of cis element in m^6^A writer genes. **(C)** Cis elements were classified into those responsive to light, phytohormones, development, environmental stress, and other regulated categories.

To gain deeper insights into the transcriptional regulatory activity of RNA m^6^A writer genes, we predicted the cis elements within the 2000-bp promoter regions of all candidates using the PlantCARE web server (http://bioinformatics.psb.ugent.be/webtools/). The identified cis-acting elements were involved in 25 functional categories, which could be classified into five groups: light-responsive elements, phytohormone-responsive elements, environmental stress-related elements, development-responsive elements, and other elements (**Figures 6A, B**). Remarkably, light-responsive elements could be identified in all promoters. Among the phytohormone-responsive elements, those involved in the abscisic acid (ABA) response and methyl jasmonate (MeJA) response were the most abundant. The most frequent environmental stress-related elements were anaerobic induction, followed by the low temperature-responsive ones. Furthermore, cis elements related to the developmental response were also identified (**Figure 6B**). The detection of light-responsive elements in all promoters (**Figure 6C**) suggests that the expression levels of m^6^A writer genes are probably influenced by light, which may affect the development and environmental stimuli responses of soybean.

### Expression patterns of *GmMTA*s and *GmMTB*s in response to abiotic stress

**Figure 7:**
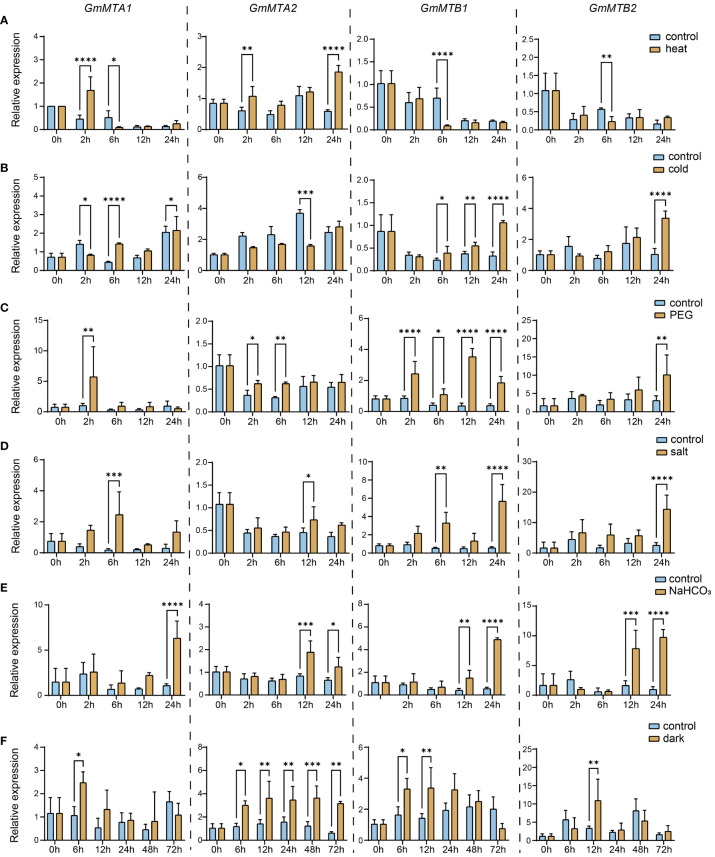
Relative expression levels of GmMTAs and GmMTBs in leaf under different abiotic stresses detected using reverse transcription quantitative PCR (RT-qPCR). 15-day-old soybean seedings were subjected to heat **(A)**, cold **(B)**, polyethylene glycol (PEG) **(C)**, salt **(D)**, alkalinity (NaHCO3) **(E)**, or darkness **(F)** treatments. GmF-BOX (Glyma.12G051100) was used as the internal control. Error bars represent SD (n = 3, *P ≤ 0.05; **P ≤ 0.01; ***P ≤ 0.001; ***P ≤ 0.0001).

The results of the cis-element analysis suggest that m^6^A RNA modifications in soybean play crucial roles in the response to different abiotic stresses. We therefore analyzed the expression pattern of the m^6^A writer components *GmMTA1, GmMTA2, GmMTB1*, and *GmMTB2* in the leaf and root of 15-day-old soybean plants grown under normal conditions or different stresses, including cold, heat, drought, salinity, alkalinity, or darkness, over 24 h. Generally, the abiotic stress treatments significantly altered the expression patterns of the *GmMTA*s and *GmMTB*s compared with the control (**Figure 7**; **Supplementary Figure S2**). Notably, each gene exhibited distinct responses to different stress conditions in the leaf; for instance, *GmMTA1* was initially suppressed after 2 h of cold treatment but induced after 6 h, whereas the expression level of *GmMTA2* remained unchanged until 12 h. By contrast, the expression levels of *GmMTB1* and *GmMTB2* were both enhanced during the cold treatment (**Figure 7B**).

In addition, we also observed that *GmMTA*s and *GmMTB*s exhibit different response times to various stresses in the leaf. The expression of all four genes was increased after 12 to 24 h of alkalinity stress (NaHCO_3_) treatment (**Figure 7E**), while darkness induced these changes after 6 to 12 h (**Figure 7F**). These data provide evidence that GmMTA- and GmMTB-mediated m^6^A modifications generally participate in the abiotic stress response in soybean. Moreover, our findings also suggest that *GmMTA2* and *GmMTB1* are the dominant genes involved in the cellular response to these environmental stressors.

### Overexpression of *GmMTA2* and *GmMTB1* in soybean enhances plant tolerance to alkalinity and darkness

**Figure 8:**
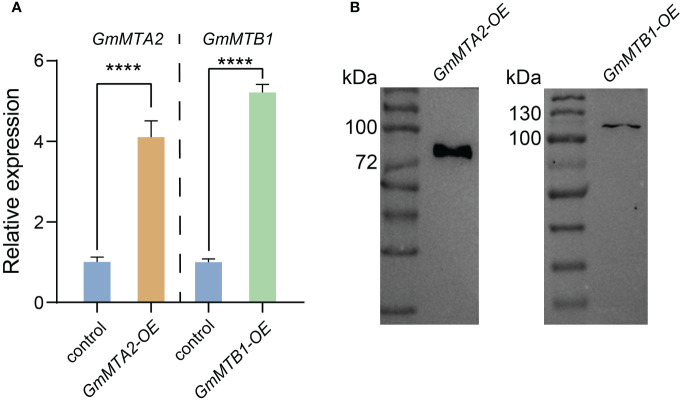
Confirmation of *GmMTA2* and *GmMTB1* overexpression in soybean. **(A)** Relative expression of *GmMTA2* and *GmMTB1* quantified using RT-qPCR in the wild type and the lines overexpressing *GmMTA2* (*GmMTA2-OE*) or *GmMTB1* (*GmMTB1-OE*). Error bars represent SD (*n* = 3, ****P ≤ 0.0001). **(B)** Immunoblot analyses of leaf extracts from the soybean lines overexpressing *GmMTA2* or *GmMTB1*. An anti-HA antibody was used for detection. Control = plants expressing the empty vector.

**Figure 9:**
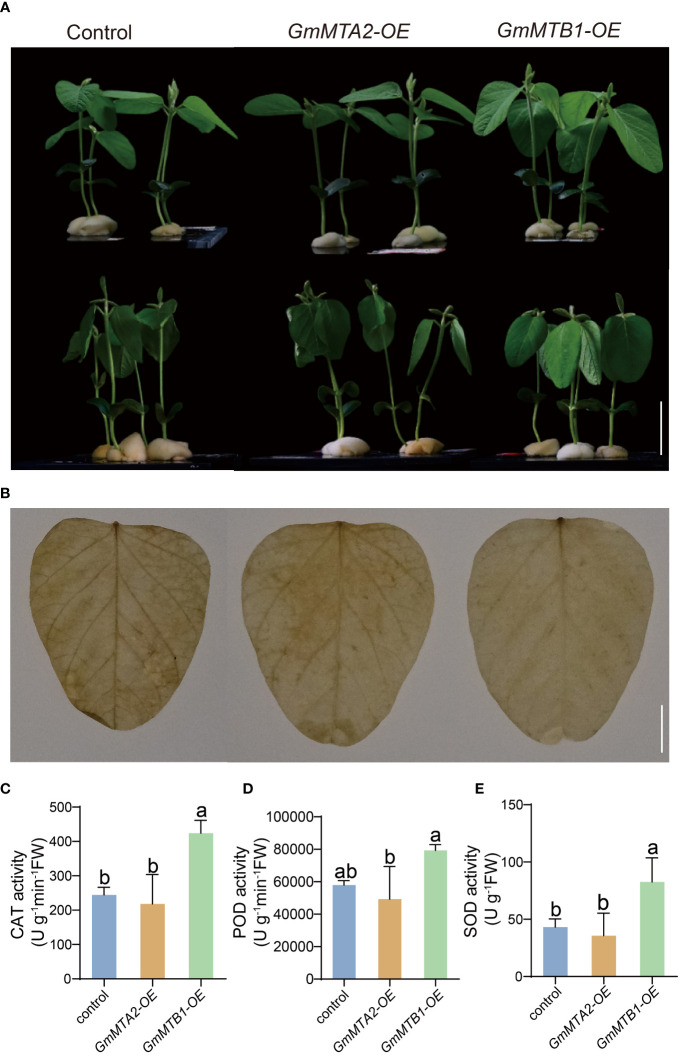
Leaf phenotypes of the empty vector*-*expressing control, *GmMTA2-OE*, and *GmMTB1-OE* plants under the NaHCO_3_ treatment. **(A)** Plant documents before (top panel) and after (bottom panel) a 36-h NaHCO_3_ treatment. Bar, 3 cm. **(B)** Diaminobenzidine staining of leaves after the NaHCO_3_ treatment. Bar, 1 cm. **(C*-*E)** The catalase (CAT; C), peroxidase (POD; D), and superoxide dismutase (SOD; E) activities in the leaves after the NaHCO_3_ treatment. Error bars represent SD (*n* = 3). Different letters indicate significant differences at *P* < 0.05.

**Figure 10:**
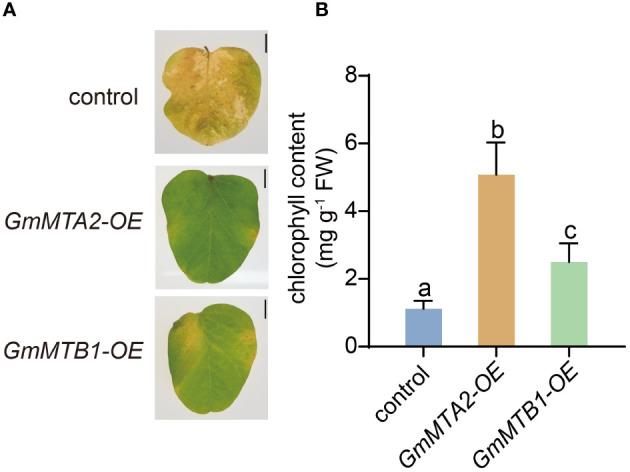
Leaf phenotypes of the control, *GmMTA2-OE*, and *GmMTB1-OE* plants upon darkness treatment. **(A)** The phenotypic changes of the leaves subjected to darkness for 10 days. Bar, 1 cm. **(B)** Chlorophyll contents of leaves from the dark-treated plants. Error bars represent SD (*n* = 3). Different letters indicate significant differences at *P* < 0.05.

To further verify the roles of GmMTAs and GmMTBs in the response to abiotic stress, we overexpressed *GmMTA2* and *GmMTB1* in the leaves of 7-day-old wild-type soybean plants, respectively, which were then subjected to alkalinity or darkness treatment. Reverse transcription quantitative PCR (RT-qPCR) and immunoblot analyses revealed that *pBA002-GmMTA2-HA* (*GmMTA2-OE*) and *pBA002-GmMTB1-HA* (*GmMTB1-OE*) were all overexpressed in the transformed soybean (**Figure 8**). Compared with the control expressing the empty pBA002 vector (EV), the leaves overexpressing *GmMTB1* but not *GmMTA2* exhibited significantly increased tolerance to the alkalinity treatment (**Figure 9A**). In addition, we detected the H_2_O_2_ content by Diaminobenzidine staining (**Figure 9B**) and measured the catalase (CAT), peroxidase (POD), and superoxide dismutase (SOD) activities (**Figures 9C-E**) in the leaves from each genotype after the NaHCO_3_ treatment. These results confirmed that the leaves harboring *GmMTB1* overexpression were indeed more resistant to the alkalinity. During the darkness treatment, the leaves overexpressing *GmMTA2* showed the highest tolerance, while leaves overexpressing *GmMTB1* also performed better than the control EV leaves (**Figure 10A**). We confirmed these findings by measuring the chlorophyll contents (**Figure 10B**). These results, together with RT-qPCR analyses (**Figure 7**), demonstrated that GmMTB1 played a role in the tolerance to alkalinity treatment while both GmMTA2 and GmMTB1 were involved in dark stress.

## Discussion

Here, we identified and characterized the gene family members of the m^6^A writer complex in legume plants. They were assigned to four families: MT-A70, WTAP, VIR, and HAKAI. In soybean, the MT-A70 family comprised five members, the WTAP subfamily had four members, and both the HAKAI and VIR families were represented by only two members each (**Figure 1**). Among these m^6^A writer candidates, the enzymatic activities of GmMTA1 and GmMTA2 were confirmed in an *in vitro* assay (**Figure 5**). In addition, the gene structures of all candidates in *G. max* were analyzed, with similar structures and conserved motifs present among the members of each family. The gene structures varied between members of the different families; however, the VIR members exhibited more exons per gene (average of 27), while members of the HAKAI family had the fewest (only three exons per gene, on average; **Figure 2**). Upon analyzing the promoter regions of the m^6^A writer complex genes, several cis elements were identified. These cis elements were subsequently categorized into five functional clusters: responsive to light, phytohormone signaling (such as MeJA and ABA), environmental stress (such as low temperature or drought), development and other regulation (**Figure 6**). Simultaneously, we performed a subcellular localization analysis of all members of the MT-A70 family from *G. max*, revealing them all to be located within the nucleus, in line with our prediction (**Table 1**).

Abiotic stress reduces crop yields and can even kill the plant; therefore, it is critical to understand how soybean responds to environmental stresses, such as saline–alkali stress, cold stress, drought stress, and darkness (Hu et al., 2021; Zheng et al., 2021). There is mounting evidence that m^6^A modifications play crucial roles in regulating plant responses to these stresses. In Arabidopsis, salt stress was reported to significantly affect the m^6^A methylation levels on mRNA (Hu et al., 2021). Additionally, VIR-mediated m^6^A methylation modulates ROS homeostasis by negatively regulating the mRNA stability of several negative regulators of the salt stress response, such as Arabidopsis NAC transcription factor (AtATAF1), GIGANTEA (AtGI), and glutathione S-transferaset U17 (AtGSTU17) (Zheng et al., 2021). In apple, MdMTA enhances lignin deposition and ROS scavenging under drought conditions. In poplar (*Populus trichocarpa*), plants overexpressing *PtrMTA* had a higher trichome density and a more developed root system (Lu et al., 2020). Moreover, they exhibited better drought tolerance (Lu et al., 2020). These findings show that the m^6^A modification plays a pivotal role in the abiotic stress responses across diverse plant species, but our understanding of these mechanisms in soybean remains very limited. Here, we identified key m^6^A component genes in soybean and analyzed their roles in different abiotic stress conditions, including cold, heat, drought, salinity, alkalinity, and darkness. Our data suggest that *GmMTA*s and *GmMTB*s, as core components of the m^6^A writer complex, are altered in response to various stressors, especially induced upon drought, alkalinity, and darkness treatment; however, their expression patterns and response times differ in accordance with the specific stress conditions (**Figure 7**).

Leaf senescence has a crucial effect on crop quality and yield. It is an age-dependent process that can be regulated by several factors, including leaf age, phytohormones, temperature, and light. Upon entering the senescence stage, a leaf’s cells undergo a sequential disorganization of cellular organelles, accompanied by systematic changes in metabolism and gene expression (Woo et al., 2019; Guo et al., 2021). Under darkness, the key transcription factors PHYTOCHROME-INTERACTING FACTOR 4/5 (PIF4/5) in the light signaling pathway are activated and regulate the expression of chlorophyll-catabolic genes through the ABA or ethylene pathways, accelerating chlorophyll degradation and promoting leaf yellowing and senescence (Sakuraba et al., 2014; Song et al., 2014). Long-term darkness has commonly been used as a tool to investigate the process of leaf senescence (Guo et al., 2021). In addition to transcriptional regulation, epigenetic modification also plays an important role in the regulation of leaf senescence. In Arabidopsis, the mutation of *MTA* resulted in a more pronounced aging phenotype than was observed in the wild type (Sheikh et al., 2024). The m^6^A levels in the mRNA were shown to increase during a darkness treatment, preventing premature aging by destabilizing transcripts of age-related genes (Sheikh et al., 2024). We assessed the expression levels of the *GmMTA*s and *GmMTB*s under a darkness treatment and observed a significant upregulation after 6-12 h (**Figure 7**). In particular, *GmMTA2* showed continuous induction and maintained high expression levels after 72 h of treatment (**Figure 7**). Furthermore, we overexpressed *GmMTA2* and *GmMTB1* in soybean leaves, which resulted in a more prominent anti-aging phenotype and higher chlorophyll content (**Figure 10**). These findings suggest that soybean GmMTA2 and GmMTB1 play essential roles in dark-induced leaf senescence, providing additional evidence for m^6^A involvement in crop or fruit ripening (Zhou et al., 2022). In animal studies related to m^6^A, METTL3 (the human homolog of MTA) is also vital for slowing the aging of human mesenchymal stem cells (Wu et al., 2020); hence, further research into the role of m^6^A in aging regulation is warranted.

## Conclusion

In this study, a total of thirteen m^6^A writer genes in *G. max*, thirteen in *G. soja*, six in *P. vulgaris*, eight in *M. truncatula*, and six in *L. japonicus* were identified. The phylogenetic analysis divided these genes into four families based on their topological structure. In soybean, the collinearity analysis revealed members from each m^6^A writer family originated from gene duplication. WoLF PSORT prediction coupled with subcellular localization analysis suggested that these m^6^A writer genes all localized in nucleus. Furthermore, enzymatic analysis showed that both GmMTA1 and GmMTA2 possessed the methyltransferase activities toward adenosine on RNA. The cis-acting elements of 2000 bp promoter regions of all m^6^A writer genes were investigated and the expression pattern of four MT-A70 family members upon abiotic stress treatment were determined. The results suggested that all members, especially *GmMTA2* and *GmMTB1*, were involved in the cellular response to various abiotic stress. Notably, soybean leaves overexpressing *GmMTB1* exhibited more resistant to the alkalinity, while overexpressing *GmMTA2* or *GmMTB1* both showed the highest tolerance to darkness treatment. These results will provide a basis for further exploring the biological functions of the m^6^A writer genes from legume plants in growth regulation and stress responses.

## Data availability statement

The datasets presented in this study can be found in online repositories. The names of the repository/repositories and accession number(s) can be found in the article/**Supplementary Material**.

## Author contributions

PL: Data curation, Formal analysis, Methodology, Writing – original draft. HL: Writing – original draft, Data curation, Formal analysis. JZ: Data curation, Formal analysis, Methodology, Writing – original draft. TY: Data curation, Writing – original draft. SG: Data curation, Writing – original draft. LC: Data curation, Writing – original draft. TX: Data curation, Writing – original draft. AX: Data curation, Writing – original draft. XL: Formal analysis, Methodology, Writing – original draft. CZ: Formal analysis, Methodology, Writing – original draft. LG: Formal analysis, Methodology, Writing – original draft. MC: Conceptualization, Funding acquisition, Investigation, Methodology, Project administration, Resources, Supervision, Validation, Writing – original draft, Writing – review & editing.
