# Supplementary material for: Genome-wide identification and functional analysis of mRNA m6A writers in soybean under abiotic stress
Source: Front Plant Sci. 2024 Jul 11;15:1446591. doi: 10.3389/fpls.2024.1446591 (PMC11269220; doi:10.3389/fpls.2024.1446591)
Supplement: Supplementary file 1 [file DataSheet_1.pdf]

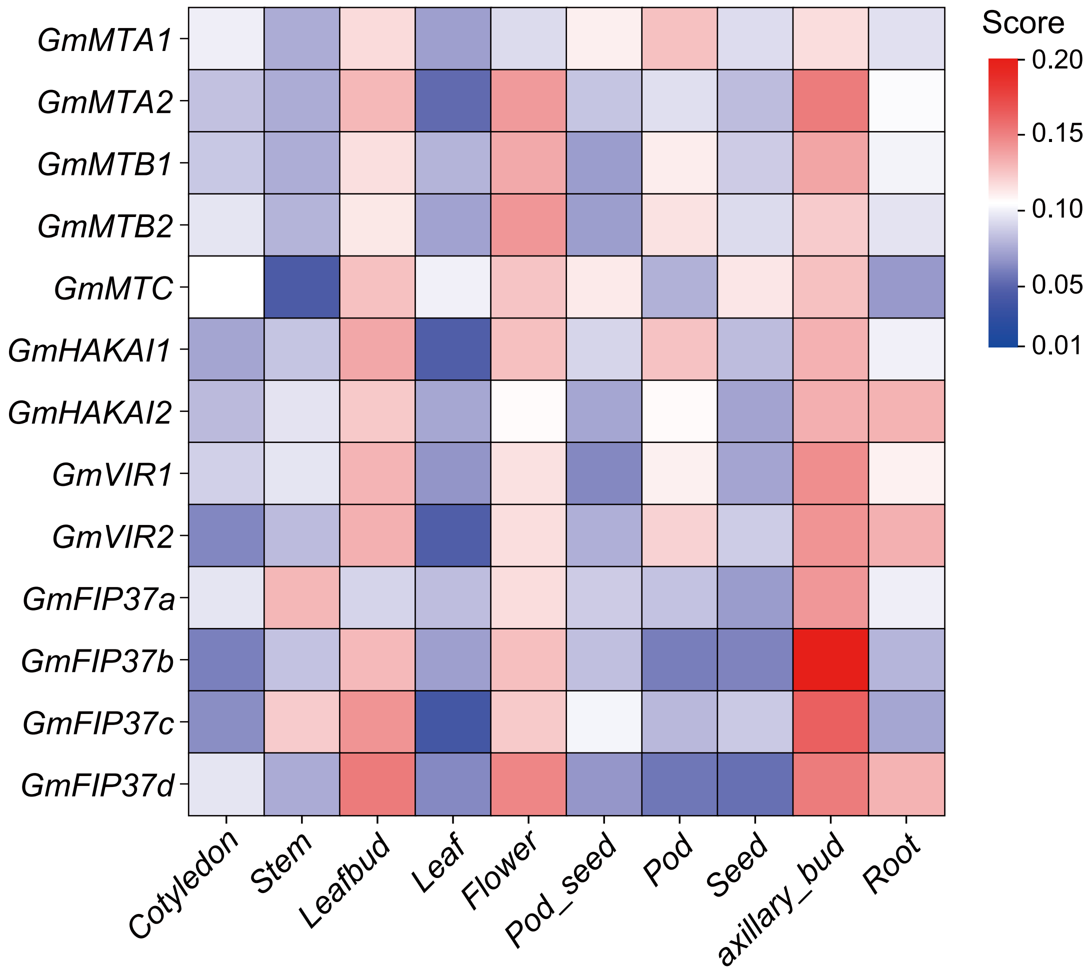

Figure S1. Expression levels of m^6A writer genes in different soybean tissues, including cotyledon, stem, leaf bud, leaf, flower, pod seed, pod, seed, axillary bud, and root, are depicted in the heatmap. The color scale bar indicates the expression levels.
