# Supplementary material for: Genome-wide identification and functional analysis of mRNA m6A writers in soybean under abiotic stress
Source: Front Plant Sci. 2024 Jul 11;15:1446591. doi: 10.3389/fpls.2024.1446591 (PMC11269220; doi:10.3389/fpls.2024.1446591)
Supplement: Supplementary file 2 [file DataSheet_2.pdf]

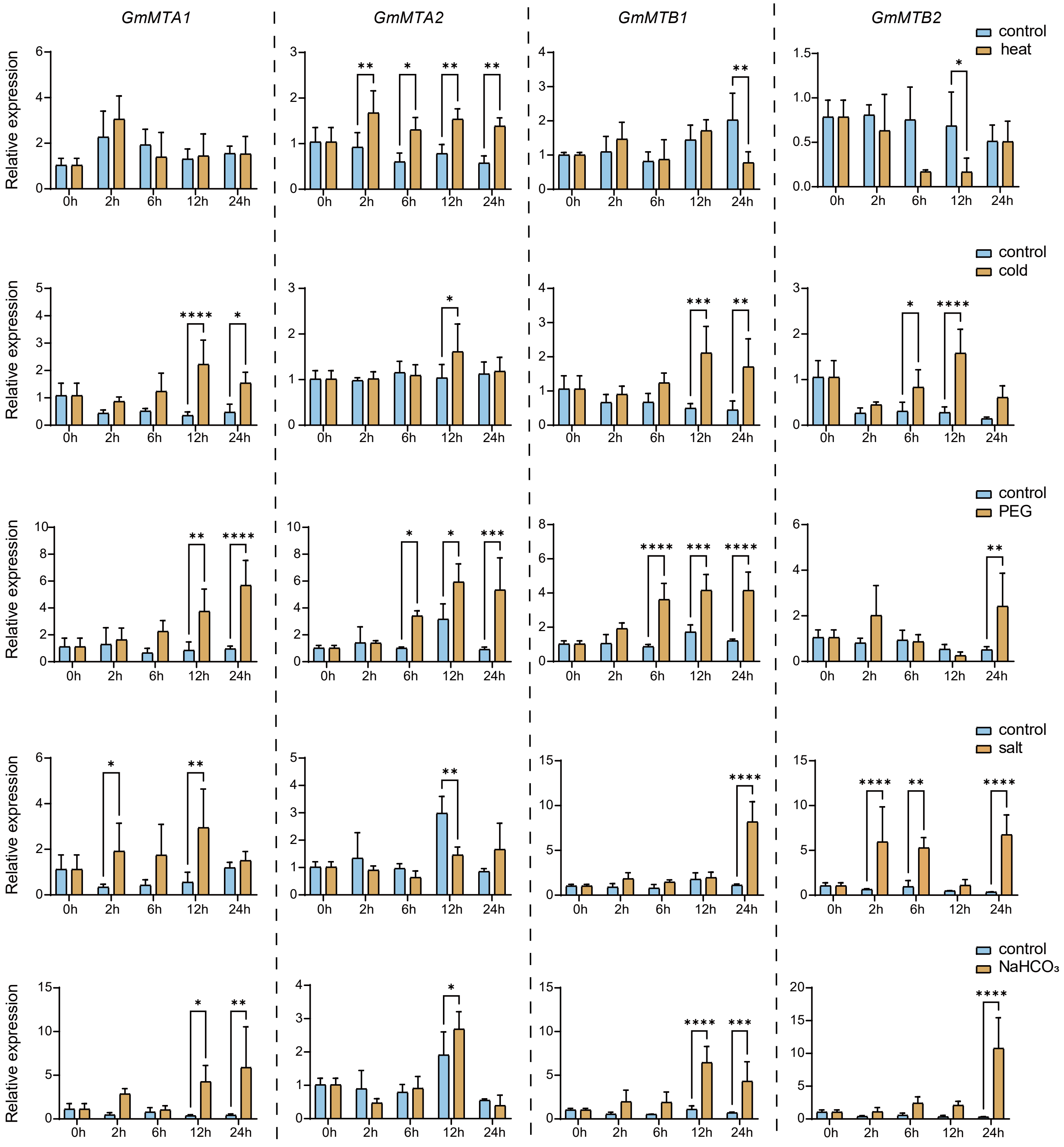

Figure S2. Relative expression levels of *GmMTA*s and *GmMTB*s in root under different abiotic stresses detected using reverse transcription quantitative PCR (RT-qPCR). 15-day-old soybean seedings were subjected to heat (A), cold (B), polyethylene glycol (PEG) (C), salt (D), or alkalinity ($NaHCO_3$) (E). *GmF-BOX* (*Glyma.12G051100*) was used as the internal control. Error bars represent SD (n = 3, *$P \leq 0.05$; **$P \leq 0.01$; ***$P \leq 0.001$; ***$P \leq 0.0001$).
