# Supplementary material for: Genome-wide identification and functional analysis of mRNA m6A writers in soybean under abiotic stress
Source: Front Plant Sci. 2024 Jul 11;15:1446591. doi: 10.3389/fpls.2024.1446591 (PMC11269220; doi:10.3389/fpls.2024.1446591)
Supplement: Supplementary file 3 [file Table_1.docx]

Supplemental Table 1. Predicted m^6^A writer candidate genes from five legume plants.

| Family | Species | Gene ID | numbers |
| --- | --- | --- | --- |
| MT-A70 | *Arabidopsis thaliana* | AT4G10760 | 3 |
|  |  | AT4G09980 |  |
|  |  | AT1G19340 |  |
|  | *Glycine max* | Glyma.07G067100 | 5 |
|  |  | Glyma.16G033100 |  |
|  |  | Glyma.10G232300 |  |
|  |  | Glyma.20G161800 |  |
|  |  | Glyma.14G077000 |  |
|  | *Glycine soja* | GlysoPI483463.16G029800 | 5 |
|  |  | GlysoPI483463.20G127800 |  |
|  |  | GlysoPI483463.14G067600 |  |
|  |  | GlysoPI483463.07G060000 |  |
|  |  | GlysoPI483463.10G195600 |  |
|  | *Phaseolus vulgaris* | Phvul.010G102500 | 3 |
|  |  | Phvul.007G073300 |  |
|  |  | Phvul.001G016200 |  |
|  | *Medicago truncatula* | Medtr8g104280 | 3 |
|  |  | Medtr1g100210 |  |
|  |  | Medtr1g021985 |  |
|  | *Lotus japonicus* | Lj3g0012917 | 3 |
|  |  | Lj5g0027810 |  |
|  |  | Lj5g0009638 |  |
| WTAP | *Arabidopsis thaliana* | AT3G54170 | 1 |
|  | *Glycine max* | Glyma.04G186400 | 4 |
|  |  | Glyma.05G040200 |  |
|  |  | Glyma.06G179400 |  |
|  |  | Glyma.17G086600 |  |
|  | *Glycine soja* | GlysoPI483463.06G165100 | 4 |
|  |  | GlysoPI483463.04G147900 |  |
|  |  | GlysoPI483463.05G036200 |  |
|  |  | GlysoPI483463.17G079600 |  |
|  | *Phaseolus vulgaris* | Phvul.002G107400 | 1 |
|  | *Medicago truncatula* | Medtr3g077320 | 2 |
|  |  | Medtr0325s0020 |  |
|  | *Lotus japonicus* | Lj4g0001447 | 1 |
| VIR | *Arabidopsis thaliana* | AT3G05680 | 1 |
|  | *Glycine max* | Glyma.02G195600 | 2 |
|  |  | Glyma.10G082100 |  |
|  | *Glycine soja* | GlysoPI483463.10G072900 | 2 |
|  |  | GlysoPI483463.02G161400 |  |
|  | *Phaseolus vulgaris* | Phvul.008G108800 | 1 |
|  | *Medicago truncatula* | Medtr1g056800 | 1 |
|  | *Lotus japonicus* | Lj5g0003460 | 1 |
| HAKAI | *Arabidopsis thaliana* | AT5G01160 | 1 |
|  | *Glycine max* | Glyma.07G144300 | 2 |
|  |  | Glyma.18G195500 |  |
|  | *Glycine soja* | GlysoPI483463.07G124800 | 2 |
|  |  | GlysoPI483463.U002000 |  |
|  | *Phaseolus vulgaris* | Phvul.007G267500 | 1 |
|  | *Medicago truncatula* | Medtr7g066710 | 2 |
|  |  | Medtr7g056183 |  |
|  | *Lotus japonicus* | Lj3g0024478 | 1 |
