# Supplementary material for: Genome-wide identification and functional analysis of mRNA m6A writers in soybean under abiotic stress
Source: Front Plant Sci. 2024 Jul 11;15:1446591. doi: 10.3389/fpls.2024.1446591 (PMC11269220; doi:10.3389/fpls.2024.1446591)
Supplement: Supplementary file 4 [file Table_2.docx]

Supplemental Table 2. Predicted conserved motif within the m^6^A writer candidates in *G. max*.

|  | Amino Acid Sequence |
| --- | --- |
| Motif1 | DKKVKELQDNIAAVNFTPQSKMGKMLMAKCRTLQEENEEIGNQASEGKMH |
| Motif2 | INYLQALKSSEESLREQLEKAKKKEAAFIVTFAKREQEIAELKSAVRDLK |
| Motif3 | ASGVTTGMILSLRESLQNCKDMLATCQNELEAAKSEIQEWH |
| Motif4 | KYQNILLRPPFEGLQKHMEGLTBDVERSNEMVLMLQ |
| Motif5 | EQGRZCLKKWGYKRVEDICWVKTNKLNRTPGLGRDGHWLFQ |
| Motif6 | PPSMQARRLLLDPAVHEEFTRLKNLVEEK |
| Motif7 | PKYFDDDYDFGGGFGGTHSGN |
| Motif8 | RKPDDMYPIJERFAPGRRKLELFGEDHNTRAGWLSLGKZLS |
| Motif9 | GNSAPGSVPPLTHKGSRVPGPPPGPGVLVPPPMPPVPPPPPDSHMNLLKV |
| Motif10 | LGKAPVEGETAKCSDNFVDAGLPVAKFIGELTLSNVVKDYPRRSELKLGE |
