# Supplementary material for: Genome-wide identification and functional analysis of mRNA m6A writers in soybean under abiotic stress
Source: Front Plant Sci. 2024 Jul 11;15:1446591. doi: 10.3389/fpls.2024.1446591 (PMC11269220; doi:10.3389/fpls.2024.1446591)
Supplement: Supplementary file 5 [file Table_3.docx]

**Primers used in this study.**

| Primer number | Sequence (5'-3') |
| --- | --- |
| Cp363 | gaatcggtgggagatttagcg |
| Cp364 | ccaaagagtgtcatcggaag |
| Cp631 | tttccgtccgcggaaacaaacacag |
| Cp632 | gcagatcgtgcgacaaaggcgagga |
| Cp633 | gattagagtgacaaggtatctggg |
| Cp634 | caatttttcatgaatctatctgg |
| Cp635 | cggagccgtgtagtgaatacaaagc |
| Cp636 | tacagagcctctagtaagacccagc |
| Cp637 | ccccgccaatataaaatggga |
| Cp638 | tccatcattgccacgggaag |
| Cp795 | gaattcctgcagcccggggactacaaagaccatgat |
| Cp796 | atcgtatggataaccccccttatcgtcatcgtcctt |
| Cp994 | gatatcgaattcctgcagcccatggagacacaatcagat |
| Cp995 | ctcgcccttgctcaccatcccgccaatgtccacatcaat |
| Cp988 | gatatcgaattcctgcagcccatggattcgagcgacagt |
| Cp989 | ctcgcccttgctcaccatccccaacagattcatttgtct |
| CP1293 | agaggatctcgaggcgcgcccatggagacacaatcagatgg |
| CP1294 | gtctttgtagtctacgtacccgccaatgtccacatcaat |
| CP1291 | agaggatctcgaggcgcgcccatggattcgagcgacagt |
| CP1292 | gtctttgtagtctacgtaccccaacagattcatttgtct |
